# A Few Features of the Drug Market

**Published:** 1907-06-29

**Authors:** 


### A FEW FEATURES OF THE DRUG MARKET.

The drug market of late has been extremely dull and uninteresting; fluctuations in value have been few and, except in the case of one or two articles, the purchaser who watches for opportunities has had little advantage over the buyer who makes his purchases to suit his needs, irrespective of market indications. There are signs, however, of renewed activity, and a careful watch should therefore be kept on the trend of events. During the last few months the quinine market has been stagnant, and although the price to which quinine sulphate has declined is low enough to tempt many people to lay in a substantial stock, there are certain important factors that should not be lost sight of; thus during the period between January 1 and May 31 of this year the Cinchona bark exported from Java to Europe amounted to no less than 7,006,000 Amsterdam pounds, which is two and a quarter million pounds in excess of the amount exported during the same period last year. The arrival of such an enormous quantity of Cinchona can hardly fail to depress the market in quinine, especially in view of the heavy stock of the alkaloid held in London at the present time. There seems, therefore, no reason to be in a hurry to buy quinine; unless something unforeseen happens the price, although already low, is more likely to recede than to advance.

### Cod Liver Oil.

Some seasons ago, in consequence of the poor catch of cod in the Norwegian fishing grounds, the value of cod-liver oil rose to the unprecedented figure of 500s. per barrel; as a consequence the consumption of this product decreased very considerably and an impetus was given to substitutes and emulsions. It frequently happened that a poor person who had been advised to take cod-liver oil was not able to afford it and, influenced by advertisements, bought instead emulsions represented to contain a large proportion of best oil; as a matter of fact these preparations were, as often as not, of very little value, containing only a small percentage of oil of the poorest quality. At the present time the finest Norwegian cod-liver oil can be bought at 70s. per barrel and, as the supply is plentiful and the demand likely to decrease as the weather gets warmer, the possibility is that the price may go a little lower.

### Camphor.

Just as the high value of cod-liver oil encouraged the use of substitutes, so the extreme rates recently ruling in the camphor market led to adulteration. All over the country dealers have been prosecuted under the Sale of Food and Drugs Act for selling camphorated oil below the standard of the British Pharmacopœia. In some cases the oil was found to contain only one-fourth the necessary amount of camphor and in one instance the sample purchased by the inspector contained no camphor at all, which meant a saving of 10s. per gallon to the dishonest trader. Since April the price of crude camphor has been steadily declining, and in forming an estimate as to the future of this article one must not lose sight of certain facts. When, a short time back, crude camphor was worth 380s. per cwt., refiners advanced their quotations to 4s. 10d. per lb. for wholesale quantities. As better supplies began to come forward from Formosa, buyers showed a disposition to await events and bought only sufficient to meet their immediate requirements. The result was that stocks increased, and at the present time it would be difficult to find purchasers at anything above 300s. per cwt. In spite of the considerable drop in the price of the crude product refined camphor remains unchanged, and it is only natural that consumers should wonder why this is the case. If refined camphor is worth 4s. 10d. per lb. when crude is quoted at 380s. per cwt., refined is dear at that price, when crude is quoted at 300s. per cwt. The probability is that refiners are waiting until their contracts are completed before reducing prices, and therefore consumers would be well advised to postpone their orders, if possible, until the expected reduction takes place. There are indications that the price of crude camphor may recede still further, for the Japanese seem to have got the upper hand of the head hunters of Formosa, who prevented the natives from working the camphor forests, and further, there is a possibility that synthetic camphor may be on the market before long. The chief requisite for the manufacture of the synthetic product is turpentine, and it is said that at the present price of turpentine synthetic camphor can be produced at 3s. 6d. per lb., but as yet it is not offered for medicinal purposes.

### Some of the Drugs.

High prices are ruling for citric acid, mainly owing to the difficulty experienced in obtaining a sufficiency of raw material from Sicily. The demand for citric acid will increase during the hot weather and an advance in price would not come as a surprise. The position of the opium market is interesting; owing to the bad weather that has prevailed in the growing districts of Smyrna there has been a shortage of supply, and prices have advanced to such an extent as to warrant manufacturers of morphine and codeine in advancing their prices several times recently. There seems no reason to expect any important change in the situation immediately. Cocaine hydrochloride is apparently steady at 9s. 4d. per oz., but it is hardly possible to foresee what changes may take place in the price of this drug; prohibitive legislation in India and the United States does not appear to have lessened the consumption to the exent expected, and in the absence of serious competition between makers there seems little reason to expect to see much lower prices for cocaine. The market for bromides remains in the same unsettled position in which it has been for the past twelve months, but sooner or later manufacturers will come to an understanding and the price of potassium bromide will advance. An advance in the price of lithia citrate would not come as a surprise. No remarkable changes have taken place recently in the prices of drugs ruled by monopolies and conventions, but as such products are not subservient to the law of supply and demand one seldom has warning of an impending change.

